# Laurate Biosensors Image Brain Neurotransmitters *In Vivo*: Can an Antihypertensive Medication Alter Psychostimulant Behavior?

**DOI:** 10.3390/s8064033

**Published:** 2008-07-04

**Authors:** Patricia A. Broderick, Helen Ho, Karyn Wat, Vivek Murthy

**Affiliations:** 1 Dept.of Physiology and Pharmacology, The City University of New York (CUNY) Medical School, Sophie Davis School of Biomedical Education, City College of New York, NY, NY, USA; 2 Doctoral Program, Dept. Biology, Dept. Psychology, CUNY Graduate School, NY, NY, USA; 3 Dept.of Neurology, NYU School of Medicine, NYU Comprehensive Epilepsy Center, NY,NY,USA

**Keywords:** anxiety, brain, caffeine, cocaine, dopamine, electrochemistry, G-protein receptor complexes (GPRC), homovanillic acid, hypertension, ketanserin, L-tryptophan, mesolimbic, motor tracts, neuromolecular imaging, nucleus accumbens, open-field behaviors, psychostimulants, serotonin, ventral tegmental area

## Abstract

Neuromolecular Imaging (NMI) with novel biosensors enables the selective detection of neurotransmitters *in vivo* within seconds, on line and in real time. Biosensors remain in place for continuing studies over a period of months. This biotechnological advance is based on conventional electrochemistry; the biosensors detect neurotransmitters by electron transfer. Simply stated, biosensors adsorb electrons from each neurotransmitter at specific oxidation potentials; the current derived from electron transfer is proportional to neurotransmitter concentration. Selective electron transfer properties of these biosensors permit the imaging of neurotransmitters, metabolites and precursors. The novel BRODERICK PROBE^®^ biosensors we have developed, differ in formulation and detection capabilities from biosensors/electrodes used in conventional electrochemistry/voltammetry. In these studies, NMI, specifically, the BRODERICK PROBE^®^ laurate biosensor images neurotransmitter signals within mesolimbic neuronal terminals, nucleus accumbens (NAc); dopamine (DA), serotonin (5-HT), homovanillic acid (HVA) and L-tryptophan (L-TP) are selectively imaged. Simultaneously, we use infrared photobeams to monitor open-field movement behaviors on line with NMI in the same animal subjects. The goals are to investigate integrated neurochemical and behavioral effects of cocaine and caffeine alone and co-administered and further, to use ketanserin to decipher receptor profiles for these psychostimulants, alone and co-administered. The rationale for selecting this medication is: ketanserin (a) is an antihypertensive and cocaine and caffeine produce hypertension and (b) acts at 5-HT_2A/2C_ receptors, prevalent in NAc and implicated in hypertension and cocaine addiction. Key findings are: (a) the moderate dose of caffeine simultaneously potentiates cocaine's neurochemical and behavioral responses. (b) ketanserin simultaneously inhibits cocaine-increased DA and 5-HT release in NAc and open-field behaviors and (c) ketanserin inhibits 5-HT release in NAc and open-field behaviors produced by caffeine, but, surprisingly, acts to increase DA release in NAc. Importantly, the latter effect may be a possible adverse effect of the moderate dose of caffeine in hypertensive patients. Thus, an antihypertensive medication is shown here to play a role in inhibiting brain reward possibly via antihypertensive mechanisms at DA and 5-HT receptor subtypes within DA motor neurons. An explanatory note for the results obtained, is the role likely played by the G Protein Receptor Complex (GPRC) family of proteins. Empirical evidence shows that GPRC dimers, heteromers and heterotrimers may cause cross-talk between distinct signalling cascade pathways in the actions of cocaine and caffeine. Ligand-directed functional selectivity, particularly for ketanserin, in addition to GPRCs, may also cause differential responses. The results promise new therapeutic strategies for drug addiction, brain reward and cardiovascular medicine.

## 1. Introduction

NMI with BRODERICK PROBE^®^ biosensors selectively detects neurotransmitters, metabolites and precursors of neurotransmitters *in vivo* in DA motor neurons which direct movement behaviors. We focused on imaging DA, 5-HT, HVA and L-TP in NAc, while simultaneously monitoring open-field behaviors. The specific open-field behaviors studied were: locomotion (forward ambulatory motion), stereotypy (repeated grooming motion, also called fine movements) and central locomotion (forward ambulatory motion in the central part of the chamber, indicating reduced anxiety). Thus, NMI provides a close cause and effect relationship between brain and behavior. Current technologies, e.g., microdialysis, are limited because microdialysis devices can traumatize brain tissue (1).

We used NMI, based on an electrochemical method of analysis, because NMI provides advantages over spectroscopic or chromatographic methods. For example, NMI (a) enhances the specificity, selectivity, simplicity and sensitivity of its spectroscopic and chromatographic counterparts, (b) does not need pre-/post-assay functional group derivatives and (c) selectively detects neurotransmitters within a complex living matrix *in vivo.*

**Figure 1.**
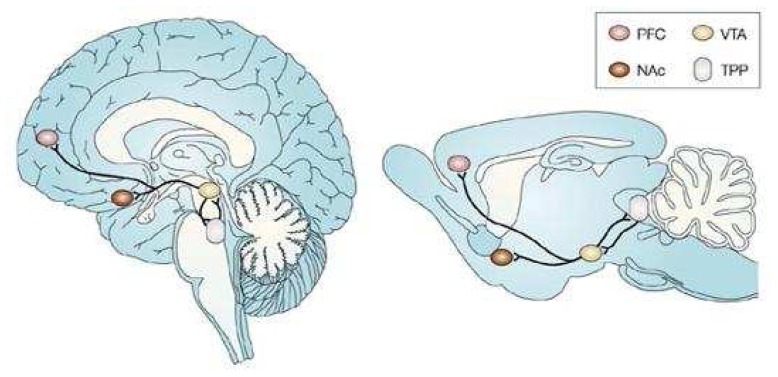
Human (left) and murine brain (right) depicting mesolimbic and mesocortical DA pathways which originate in VTA and send ascending projections to NAc and Prefrontal Cortex (PFC). Feelings of reward as well as aversion are derived herein. VTA sends descending projections to the tegmental pedunculopontine nucleus (TPP), implicated in non-DA mediated reward signalling. *(Adapted by permission from Macmillan Pub. Ltd., NatureReviews Neurosci. 2004, 5, 55-65; http://www.nature.com/nrn/journal/v5/n1/fig_tab/nrn1298_F2.html)* (89).

**Figure 2.**
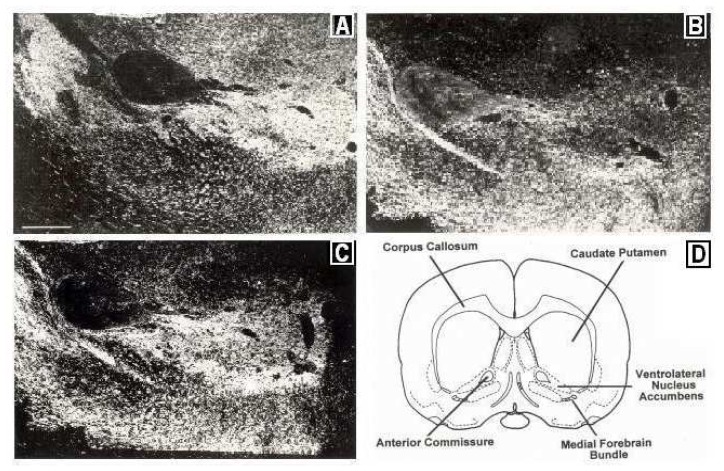
Immunocytographs of DA and 5-HT in NAc (ventrolateral (vl)) of Sprague Dawley laboratory rats. Dark field photomicrographs show the distribution of (A) DA neurons, stained with tyrosine hydroxylase; two high density patterns of DA are apparent in the medial and lateral core, (B) 5-HT axons in the caudal one-third of NAc; 5-HT was stained with a sensitive silver intensification procedure, thus axons and terminals are black, (C) 5-HT axons in DA neurons in NAc at the site of the BRODERICK PROBE^®^ laurate biosensor. In B, two low density patterns of 5-HT are apparent in the ventral and ventrolateral NAc. High density 5-HT is seen in the perimeter around the core. (scale-horizontal line shown in bottom left of Fig. 2A=500 um)(*Adapted by permission from Elsevier, Brain Research Bull., 1995, 37, 37-40)* (2). Direct efferent neurons derive from VTA to vlNAc (90). (D) Coronal section of NAc depicting vlNAc (*Adapted by permission from Springer, A Stereotaxic Atlas of Rat Brain, 1979, 20)* (35).

The precise focus of our studies in brain is the mesolimbic pathway in the freely moving (unrestrained) and behaving animal *in vivo*. Figure 1 depicts schematically the mesolimbic neuronal circuit in brain. We collaborated with Dr. Clyde Phelix, San Antonio, Texas, to perform immunocytochemical studies that show a significant overlap in the presence of DA and 5-HT in DA axons in NAc at the site of the BRODERICK PROBE^®^ biosensor (2). Figure 2 shows the immunocytochemistry results. It is important to note here that serotonergic cells in 5-HT cell bodies in dorsal raphe project axons to NAc; these axons play a critical role in the DA mesolimbic pathway to neuromodulate movement behavior (3).

The aims are to use NMI, BRODERICK PROBE^®^ laurate biosensors and infrared photobeams to (a) study *in vivo* integrated neurochemistry and behavior produced by cocaine and caffeine alone and co-administered and (b) study the effects of the antihypertensive medication, ketanserin, on cocaine and caffeine responses alone and co-administered.

Cocaine is known to be a reinforcer of psychostimulant behavior (4). Cocaine increases DA reuptake inhibition and DA release at the synapse in mesolimbic and nigrostriatal brain reward centers, thereby inducing a feeling of “joie de vivre”. Cocaine enhances brain reward by pharmacologic sensitization, i.e., repeated use causes enhanced reward in part, *via* adenosine inhibitors (5,6). Nonetheless, cocaine produces neuroadaptive withdrawal symptoms and hypertension (4,7).

Caffeine is known to reinforce psychostimulant behavior, increase DA concentrations in mesolimbic and nigrostriatal pathways in brain and has adenosine receptor inhibiting properties (8). Similarly to cocaine, caffeine produces withdrawal symptoms and hypertension (9). Unlike cocaine, caffeine produces pharmacologic tolerance, i.e., repeated use of caffeine causes diminished responses (9).

Moreover, unlike cocaine, whose only clinical use is as a local anesthetic, caffeine has been shown to have several clinical and possible clinical uses. Caffeine may reduce traumatic brain injury (TBI) in animals (10). Caffeine is used clinically to assist breathing in preterm babies and to increase alertness and performance in adult patients (11). Recent discoveries about caffeine include a report that caffeine may reduce risk of diabetes by 50% (12). Caffeine may protect against Parkinson's Disease (13).

Ketanserin is an antihypertensive medication (14). Ketanserin binds with (a) 5HT_2A/2C_ receptors, (b) histamine, (c) adrenergic (α_1_) receptors, (d) 5-HT_1A_ receptors in the human (pK*_i_* value, 5.9 and 6.2), (e) 5-HT_2B_, adrenergic α_2_ receptors and (f) acetylcholine (Ach) muscarinic receptors. Ketanserin does not bind to DA receptors (15, 16). In a clinical study, ketanserin blocked adenosine-induced bronchospasm, providing a possible link between ketanserin and adenosine receptors (17). We used ketanserin to delineate receptor profiles for cocaine and caffeine because this medication selectively binds to 5-HT_2A/2C_ receptors, with high affinity, in mesolimbic areas of brain.

## 2. Methods

### 2.1. Conventional Voltammetry and Microvoltammetry

Significant advances in voltammetry and microvoltammetry have been made with the use of carbon fiber and carbon paste biosensors. For example, Nafion^®^ a perflourosulfonated compound, as well as ascorbic acid enzyme inhibitors are used as coatings on biosensors to separate ascorbic acid from DA. Also, a subtractogram method is used for interpreting *in vivo* recordings to separate 5-HT from DA. Therefore, brain neurotransmitter studies *in vivo* have been in progress for quite some time (cf. 18,19 for reviews of the literature).

Electrochemical methods:

- Involve measurement of current as a function of applied potential, wherein
- electroactive species undergo redox reactions at a characteristic redox potential.
- Formula:O + ne^−^ ⇔ R, wherein, ne = number of electrons, O=oxidation, R= reduction.
- The current that is produced by a specific redox reaction is proportional to the concentration of neurochemicals, according to the Cottrell equation, described below.

Historically, methods based on conventional voltammetry and microvoltammetry, as pioneered in the 1970's, have validated that the flow of charge, *i.e.*, amount of current in amperes, which passes through the surface of an indicator electrode is proportional to the concentration of the electroactive species studied (18-20). The following formulas describe this relationship in terms of charge, electron transfer, current, diffusion layer, time, Faraday's constant, size of the indicator electrode and concentration (mass) of electroactive species.

$$\begin{array}{ccc} Q={\text{nFVC}}_{oR} & \;i=dQ/dt & \;i=\text{nFV}\;dC_{R,t}/dt \end{array}$$

where *V* is the volume of the diffusion layer on the electrode where the measurement is being made, *n* is the number of electrons transferred, *F* is the Faraday Constant, and *C_o_* denotes initial concentration. The Cottrell equation is derived from the formulas written above and demonstrates that current i.e., charge and mass, i.e., concentration, are proportional. The Cottrell equation is:

$$i_{t}={\text{nFAC}}_{o}{D_{o}}^{1/2}/3.14^{½}\;{\text{t}}^{½}$$

where:

o=concentration of electroactive species oxidized.
i= current at time, t
n= number of electron transfers, eq/mol
F= Faraday's constant, 96486 C/eq
A= electrode area, cm ^2^
C= concentration of o, mol/cm^3^
D= Diffusion coefficient of o, cm^2^/s

### 2.2. Neuromolecular Imaging (NMI)

NMI has made significant advances in the field of electrochemical methods. Specifically, (a) formulations and detection capabilities of biosensors are different. We embedded a series of saturated and unsaturated fatty acid and lipid surfactant assemblies into carbon-paste-based biosensors in a variety of concentrations to allow advanced detection capabilities e.g., selective imaging of ascorbic acid, DA, 5-HT, HVA, L-TP and peptides, such as dynorphin and somatostatin (21-26), (b) with NMI biosensors, there is no need for cumbersome head stages as are needed by conventional *in vivo* voltammetric and microvoltammetric methods (27,28) because NMI biosensors have low resistance properties, (c) NMI biosensors are resistant to bacterial growth (26), (d) Unlike carbon fiber biosensors, NMI biosensors do not form gliosis, i.e., scar tissue which impedes detection of neurotransmitters, causing electrochemical signals to decay (29) and (e) Like other carbon-paste-based biosensors, NMI biosensors respond to the lipid matrix of the brain by enhancing electron transfer kinetics; this property improves the sensitivity, selectivity and operational stability of the biosensors, allowing the detection of reliable electrochemical signals that are long-lasting (29).

**Figure 3.**
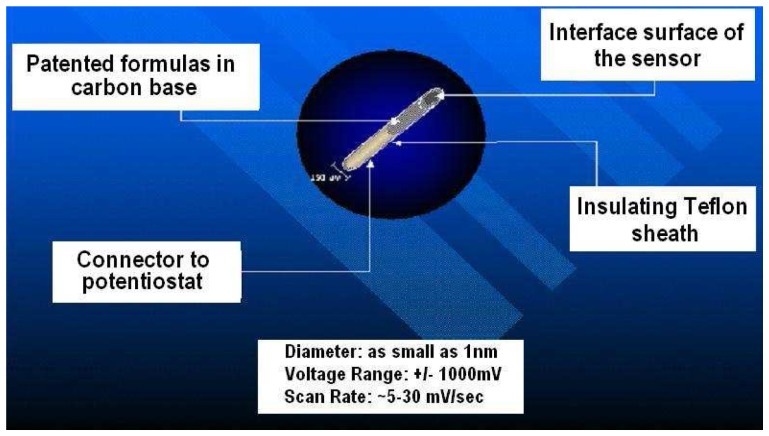
Schematic of the generic BRODERICK PROBE^®^ biosensor with specifications.

In this paper, results from NMI laurate biosensors are presented. Lauric acid has a hydrophobic head, hydrophilic tail, and acts as a surfactant to reduce surface tension. The surfactant, lauric acid, also acts to assist the migration of molecules to form an oriented, adsorbed film on the interfacial surface of the indicator sensor. This mechanism is a key characteristic for electron transfer kinetics exhibited by NMI biosensor subtypes. Figure 3 shows a schematic diagram of a BRODERICK PROBE^®^ biosensor with specifications.

### 2.3. Manufacture of Laurate Biosensors

Laurate biosensors are manufactured on site. Lauric acid is a saturated fatty acid (formula: CH_3_(CH_2_)_10_COOH). Laurate biosensors, for these studies, were comprised of < 1 mg aliquot of stock, a mixture of ultra-pure carbon (1.5g), oil (Nujol) containing alpha tocopherol (1.24 g), and lauric acid (100 mg). Details of the construction of the laurate biosensor are published (21-25).

### 2.4. NMI Properties of Laurate Biosensor

Oxidation Potentials for these biosensors, *in vitro*, are, in the order of detection from lowest to highest positive potential, ascorbate [0.08 V]; DA [0.13 V]; 5-HT [0.29 V]; HVA [0.43V] and L-TP [0.63]. Oxidation Potentials for these biosensors, *in vivo*, are, in the order of detection from lowest to highest oxidation potential, ascorbate [0.09 V], DA [0.14 V], 5-HT [0.31 V]; HVA [0.44V] and L-TP [0.64]. Each specified oxidation potential is within a range of +/− 0.015V.

### 2.5. Semiderivative Circuit

During the performance of research in the electrochemical analysis and biosensor field, this laboratory has studied and improved electrical circuits for imaging a multifaceted array of neurochemicals. Although NMI biosensors can be used with many different voltammetric electrical circuits, we primarily, but not exclusively, have worked with one which semidifferentiates/semiderivatizes the voltammetric linear ramp circuit. Importantly, we discovered that a reduction semiderivative circuit is optimal for oxidizing neurochemicals for this type of analysis (22). Further, by studying a variety of capacitors and resistors for use with potentiostats and miniature biosensors, we found that either a one second or a five second time constant is optimal for imaging neurotransmitters *in vivo*.

### 2.6. Calibration Procedures

Biosensors are precalibrated *in vitro* in a freshly made deoxygenated physiological saline-phosphate buffer solution (0.01 M, pH=7.4 containing nmol aliquots of DA, 5-HT, norepinephrine (NE) (99% purity, Sigma-Aldrich, St. Louis, MO) and several other neurochemicals such as ascorbic acid, uric acid, 3,4, dihydroxyphenylacetic acid (DOPAC), HVA, L-TP and peptide neurotransmitters, such as dynorphin and somatostatin. The deoxygenation process takes place by purging the electrochemical cell with ultra pure nitrogen gas at 10 psi to remove traces of oxygen from air for one minute before the biosensor is set to cell mode at the resting potential of 0.2V before anodic scanning begins. For cell deposition/equilibration, we allow the resting potential to remain at 0.2 V for two minutes. The E1 initial potential, resting potential of 0.2 V, is selected so that during the scanning process, non-faradaic, charging current is recorded before cathodic and anodic images appear at experimentally derived characteristic oxidation potentials. Biosensors are postcalibrated *in vitro* in a fresh deoxygenated physiological saline-phosphate buffer solution, (0.01M, pH=7.4).

**Figure 4.**
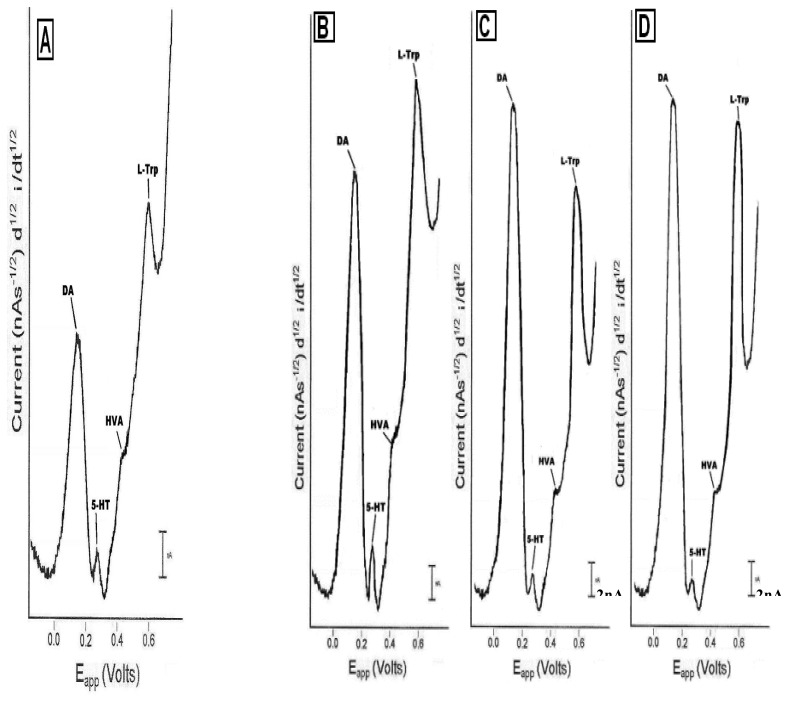
**Figure 4A** NMI recordings of neurotransmitters in NAc, drawn from raw data of baseline DA, 5-HT, HVA and L-TP signals. Figure 4B, C, and D. NMI recordings of neurotransmitters, drawn from raw data of DA, 5-HT, HVA and L-TP signals after (B) caffeine, (C) ketanserin and caffeine and (D) ketanserin, cocaine and caffeine. The y axis shows current changes in nanoamperes (nA) from baseline. The x axis shows applied potential in subunits of Volts (millivolts). Each neurochemical exhibits electron transfer properties at a specific oxidation potential. Current is proportional to the concentration of each neurochemical as described by the Cottrell Equation (18-20).

Biosensors can be lipid amplified with phosphotidylethanolamine (PEA) (10% w/v) and bovine serum albumin (BSA) (1% w/v). This process takes place for thirty min after which biosensors are immersed in saline (0.16 M) for 45 min, and finally biosensors are immersed in saline (0.16 M) phosphate (0.01M) buffer (pH 7.4). Results from lipid amplification of laurate biosensors correlates with that exhibited by electrodes used to study lauric acid by Raman Spectroscopy (30). Figure 4A depicts baseline *in vivo* neurochemical images detected by the laurate biosensor. Figures 4 B, C and D depict *in vivo* neurochemical images **detected by the** laurate biosensor after drug(s).

*In vitro* images of neurochemicals using the laurate biosensor are published (31).

### 2.7. Interpretation of NMI Signals

Dopamine and 5-HT have amine groups that are protonated at neutral pH and exist as cations; metabolites of the monoamines are not protonated at physiological pH and exist as anions (32). Cations are imaged in these studies *in vitro* and *in vivo* and anions are detected generally at higher concentrations *in vitro* and *in vivo.* Detection of anions does not interfere with that of cations because each neuromolecule is oxidized at an empirically-derived, specific oxidation potential. Calibration curves for DA, 5-HT, L-TP, norepinephrine (NE) and ascorbic acid (AA) are published with an extensive treatise on selection separation of these compounds from one another (33,34).

### 2.8. Animals and Surgical Procedures

These studies are approved by the National Institutes of Health (NIH) in accordance with the Institutional Animal Care and Use Committee (IACUC) of The City College New York, The City University of New York. To begin these studies, we purchase male, Sprague Dawley, Caesarean-derived, virus-free, laboratory rats (*rattus norvegicus*) from Charles River Laboratories, Kingston, NY. When the animals arrive at our facility, they are housed in our Marshak Vivarium for about one week before surgery begins to allow animals to become acclimated to their environment. Animals are fed Purina Rat Chow and water *ad libitum*. A twelve hour dark-light cycle is maintained both in the Vivarium and in Dr. Broderick's research laboratory where studies take place, to maintain animals' circadian rhythms.

Surgery begins with an intraperitoneal (ip) injection of the anesthetic pentobarbital Na (50 mg/kg in a dilute 6% solution)). Laurate biosensors are inserted stereotaxically (Kopf Stereotaxic, Tujunga, CA) within NAc (AP= +2.5; ML= +2.6; DV= -7.3) (35) and a Ag/AgCl microreference and stainless steel microauxiliary are placed in contact with dura. Indicator laurate biosensors are held in place with Splintline Acrylic (Lang Dental, Il.). Temperature is continuously monitored with a rectal probe and thermometer (Fisher Sci., Fadem, NJ); temperature is maintained at 37.5°C ± 0.5°C with an aquamatic K module heating pad (Amer. Hosp. Supply, Edison, NJ). Pinnal, corneal and leg flexion responses are monitored throughout surgery and supplemental doses of Na pentobarbital are administered to maintain adequate pharmacokinetic induction and depth of anesthesia. Physiologic saline (animal body weight in cc's) is injected at the completion of surgery to maintain proper electrolyte and volumetric status. The total time for surgery is two to three hrs. Animals are housed individually after surgery and recover from surgery with food and water *ad lib* before the experimental studies begin.

### 2.9. Neuromolecular Imaging (NMI) On Line with Open Field Behavior

Studies are performed in freely moving (unrestrained) animals during movement behavior. Each animal is placed in a Plexiglas^®^ faradaic chamber insulated from white light frequency with a copper sheath (dimensions: 24″ × 18″ × 23.5″). The three-biosensor assembly is connected to a CV37 potentiostat (BAS, West Lafayette, IN) or a computerized Autolab Analyzer (manufactured at EcoChemie, Netherlands and distributed by Brinkmann, Westbury, NY). NMI signals are transduced to a mercury commutator. (Br. Res. Instr., Princeton, NJ) by a flexible cable, and a mating connector (BJM Electronics, Staten Island, NY). Cables are designed and manufactured on site. The potentiostat is electrically connected to a Minigard surge suppresser (Jefferson Electric, Magnetek, NY) which is connected to an electrical ground in isolation. When the CV37 potentiostat is used, NMI is recorded on a strip chart recorder (The Recorder Company, San Marcos, TX). Physiologic steady state for electrochemical signals is achieved before drug(s) are administered. Neurochemical data are normalized to 100% of control/baseline values.

**Figure 5.**
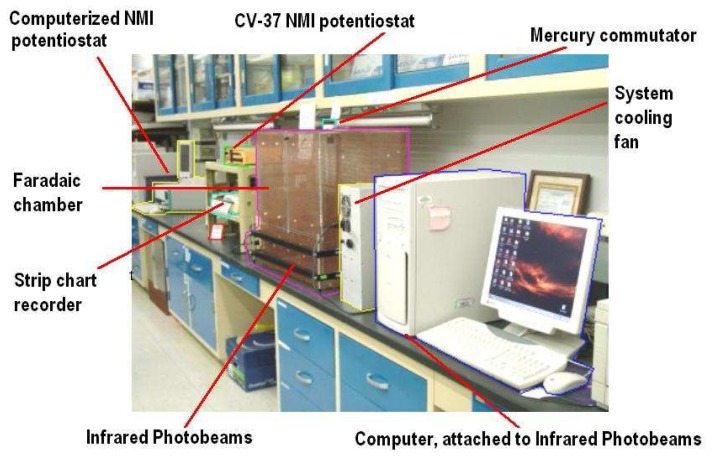
Digital photographs of the NMI analytical devices and behavioral (open-field) equipment used in the Broderick laboratory for the simultaneous study of brain and behavior.

NMI signals are imaged *at the same time* as open-field behaviors are monitored. Figure 5 shows the NMI electro-analytical and behavioral device set-up, designed on site. The faradaic chamber in which the NMI signals are recorded, is fitted with infrared photobeams set into aluminum frames, placed 3/4 inches above the chamber's floor. The infrared photobeams are activated by a Pentium 3 circuit using a modified version of the Activity Pattern Monitor (APM) (San Diego Instruments, San Diego. CA). The system permits simultaneous and selective monitoring of movements in real time at a resolution 1.5 inches in 0.1 secs. The animal's position is based on X-Y axes calculated in terms of one of 16 equally sized sectors and one of nine unequally sized regions. Every 100 msec, the computer checks the status of all the beams in the chamber. Behaviors are recorded as frequency of photobeam crossings.

Locomotion (Ambulation) is described as continuous movement in a forward direction. Stereotypy is described as repeated grooming motions or Fine Movements. Central locomotion is described as movements into the center of the chamber which provides evidence of a reduction in anxiety because rodents usually move around the corners and walls of an enclosure during natural movement. Thigmotaxis is the term used for corner, wall behavior. Thigmotaxis is used as a test for indicating that the animal is feeling anxiety. Increases in central locomotion behavior reliably determine anxiety reduction (36-38). Enhanced anxiety is displayed by animals who have a diminished desire to explore novel environments (39).

### 2.10. Study Design

NMI electrochemical signals for neurochemicals are recorded separately at distinct oxidation potentials, within seconds and sequentially. Recordings are repeated every five min for a period of 2 hrs before drug(s) are administered. The first hr pre-drug, allows exploratory behavior in a novel environment. The second hr pre-drug allows the animal to become habituated before drug(s) are administered. Three points, at the end of the habituation period, at which time the animal no longer responds to external stimuli, provides the baseline for both neurochemical and behavioral data.

### 2.11. Confirmation of Biosensor Placement

Placement within NAc is confirmed by the potassium ferrocyanide blue dot method (specifications: current in mA, 30; time in sec, 40).

### 2.12. Statistics

One-Way Analysis of Variance (ANOVA) as studied by the PRISM program, is used to determine statistically significant differences in neurochemical and behavioral responses after drug(s) administration within and between the major groups studied. Statistical analyses within each individual group are performed by Tukey's *post hoc* tests. Alpha significance is set at the P=0.05 level.

## 3. Results and Discussion

### 3.1. Changes in neurochemicals and behavior after cocaine

*“Why do people use cocaine?”* To say that addiction is a complex and poorly understood behavior is an understatement. Addiction is a compulsive disorder which is maintained despite tremendous adverse consequences. At least, recent discoveries in the field of cocaine addiction have provided some answers to this question, “why…? ” (cf. 4, 40 for reviews). Some of these recent discoveries are: (a) females are more responsive to cocaine than are males; these data have been reported in animals and humans (41,42), (b) the reinforcing properties of cocaine can lead to a psychosis similar to a schizophrenic psychosis in animals and humans; the atypical antipsychotic medication, risperidone, may be a useful therapy (43), (c) serotonin plays an important role in the reinforcing effects of cocaine because 5-HT is known to neuromodulate DA, which is the primary reinforcer; these data are from animal studies (44,46) and (d) further animal studies show that there is an adenosine involvement in the cocaine reactions (6,48). More answers from clinical studies are forthcoming. In fact, possible pharmacotherapies for cocaine addiction, based on DA (45) 5-HT (46), DA transporter antagonists (47) and adenosine/DA coupled GPRC heteromers (48), are in progress.

**Figure 6.**
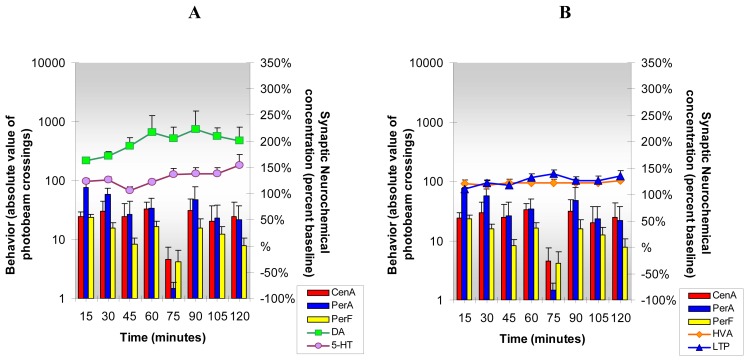
**Figure 6A and B** shows the neurochemical and behavioral changes after **acute cocaine** administration (5 mg/kg, ip). ***Statistical Significance*: Neurochemical**: Anova: all groups; p<0.005 to p<0.0004. Tukey's *post hoc* analysis: DA, 5-HT and HVA increased above baseline (p<0.001), L-TP increased above baseline (p<0.01). **Behavioral**: Anova: all groups; p<0.0042 in first hr only; Tukey's *post hoc* analysis: CenA increased above baseline (p<0.01), PerA and PerF increased above baseline (p<0.001).

The results from the present studies, Figure 6A and B, show that acute cocaine increases DA, 5-HT, HVA and L-TP release in NAc of freely moving and behaving animals. Simultaneously, cocaine increases locomotion and stereotypy and reduces anxiety behavior. The data indicate that cocaine increases neurotransmitter turnover as well as release of neurotransmitters, metabolites and precursors in NAc. This NMI neurochemical and behavioral paradigm enables new insights into psychostimulant-induced reward-related behaviors driven by reward-related brain neurotransmitters.

**Figure 7.**
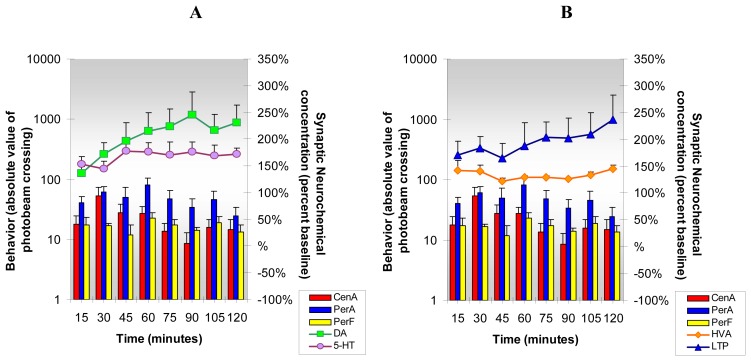
**Figure 7A and B** shows the neurochemical and behavioral changes after **acute caffeine** administration (25 mg/kg ip). ***Statistical Significance*: Neurochemical:** Anova: all groups; p<0.01 to p<0.0004; Tukey's *post hoc* analysis: DA increased above baseline (p<0.01), 5-HT and L-TP increased above baseline (p<0.001); HVA increased above baseline (p<0.05). **Behavioral:** Anova: all groups; p<0.0001; Tukey's *post hoc* analysis: CenA and PerA increased above baseline (p<0.01); PerF increased above baseline (p<0.001).

**Figure 8.**
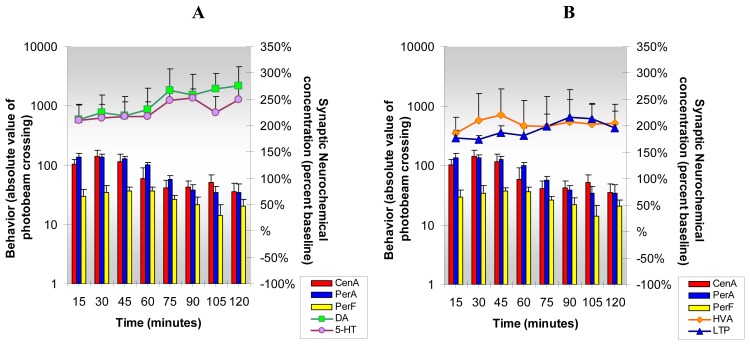
**Figure 8A and B** shows the neurochemical and behavioral actions of **co-administered cocaine and caffeine** (5 mg/kg, ip and 25 mg/kg, ip, respectively). ***Statistical Significance*: Neurochemical**: Anova: all groups; p<0.0001 to p<0.0004; Tukey's *post hoc* analysis: DA, 5-HT, HVA and L-TP increased above baseline (p<0.001). All neurochemicals in this group were significantly greater than those produced by cocaine, DA (p<0.05), 5-HT, HVA, and L-TP (p<0.001). Also, 5-HT, HVA and L-TP responses in this group were significantly greater than those produced in the caffeine group (p<0.001). **Behavioral:** Anova: all groups; p<0.0003; Tukey's *post hoc* analysis: CenA, PerA and PerF increased above baseline (p<0.001). All behaviors were significantly greater than those produced by cocaine alone (p<0.001),(p<0.05)&(p<0.01) respectively.

**Figure 9.**
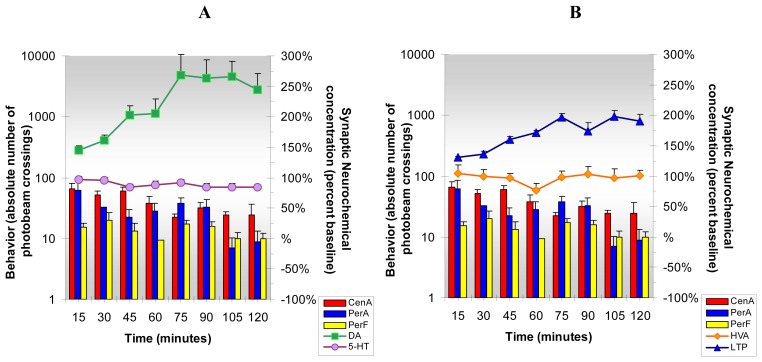
**Figure 9A and B** shows the neurochemical and behavioral effects of **acute ketanserin** administration. ***Statistical Significance*: Neurochemical:** Anova: all groups; p<0.006 to p<0.0002; Tukey's *post hoc* analysis: DA and L-TP increased above baseline (p<0.001), 5-HT and HVA increased above baseline (p<0.05). DA and 5-HT responses were similar to those of cocaine in the second hr. **Behavioral:** Anova: all groups; p<0.0082; Tukey's *post hoc* analysis: behavioral responses were unremarkable; none of the three behaviors (CenA, PerA and PerF) reached statistical significance over baseline. Data were similar to that produced by ketanserin when administered in combination with caffeine. CenA for ketanserin were similar to that of the ketanserin/cocaine group.

**Figure 10.**
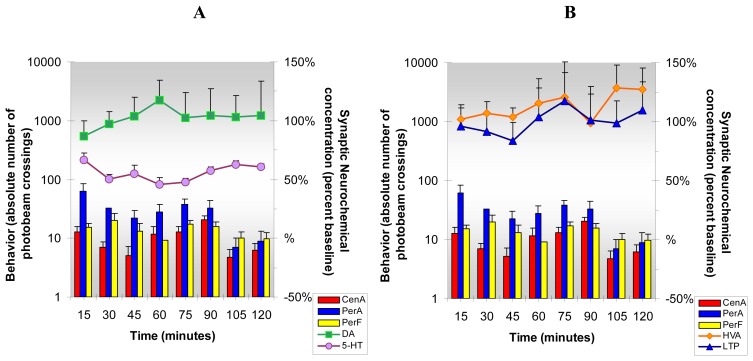
**Figure 10A and B.** shows the neurochemical and behavioral effects of **ketanserin and cocaine** (3.0 mg/kg, sc and 5 mg/kg, ip, respectively). ***Statistical Significance*: Neurochemical:** Anova: all groups; 5-HT decreased (p<0.0005); Tukey's *post hoc* analysis: DA, HVA and L-TP showed an insignificant change from baseline, 5-HT significantly decreased from baseline (p<0.001). Neurochemicals were reduced to baseline compared with cocaine alone i.e., DA and 5-HT (p<0.001) and L-TP (p<0.05). HVA was reduced compared to cocaine in the first hr. **Behavioral:** Anova: all groups; p<0.006; Tukey's *post hoc* analysis: CenA increased above baseline (p<0.01). PerA and PerF increased above baseline (p<0.001). CenA, previously produced by cocaine, were inhibited by ketanserin whereas PerA were inhibited by ketanserin only in the first hr. Ketanserin was unable to inhibit PerF produced by cocaine.

**Figure 11.**
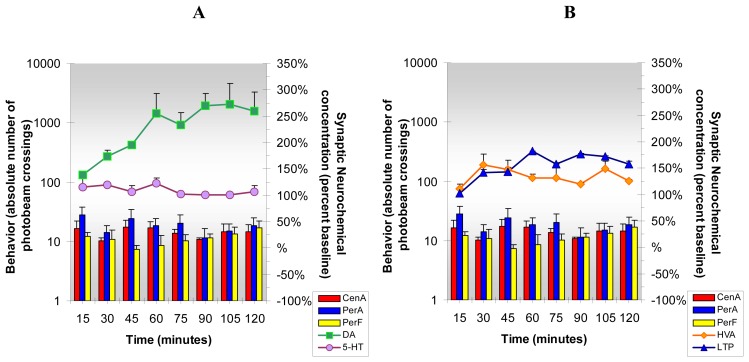
**Figure 11A and B** shows the neurochemical and behavioral effects of **acute ketanserin and caffeine** (3.0 mg/kg, sc and 25 mg/kg, ip, respectively). ***Statistical Significance*: Neurochemical:** Anova: all groups: p<0.03 to p<0.008; Tukey's *post hoc* analysis: DA increased above baseline (p<0.01), 5-HT and HVA increased above baseline but effects were not significant, L-TP Increased above baseline (p<0.05). Compared with caffeine alone, there were significant decreases in 5-HT (p<0.001) and L-TP (p< 0.01). **Behavioral:** Anova: all groups: p<0.0001; Tukey's *post hoc* analysis: PerF increased over baseline (p<0.01); CenA and PerA effects were not significant. Compared with caffeine results, all three behaviors were significantly inhibited by ketanserin (p<0.01).

**Figure 12.**
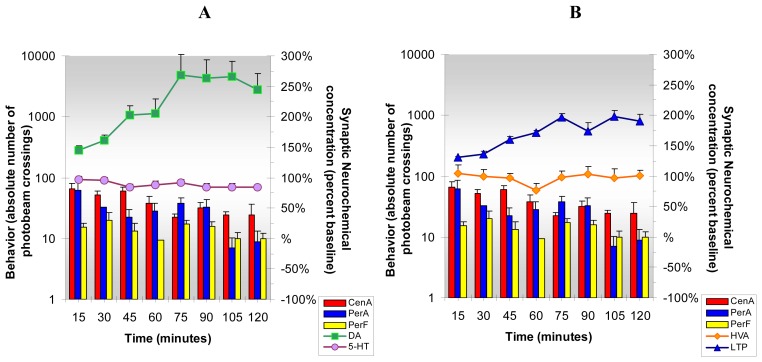
**Figure 12A and B** shows the neurochemical and behavioral effects of **acute ketanserin, cocaine and caffeine** (3.0 mg/kg, sc, 5 mg/kg, ip and 25 mg/kg, ip, respectively). ***Statistical Significance*: Neurochemical:** Anova: all group: p<0.14 to p<0.0006; Tukey's *post hoc* analysis: DA increased above baseline (p<0.01), L-TP increased above baseline (p< 0.05), 5-HT and HVA decreased to below baseline, not significant. Compared with caffeine, 5-HT and HVA were inhibited by ketanserin (p<0.01) (p<0.05 respectively). Compared with cocaine, 5-HT and HVA were also inhibited by ketanserin (p<0.001). However, L-TP is significantly higher in this ketanserin, cocaine and caffeine group compared with the cocaine group (p<0.001). **Behavioral:** Anova: all groups: p<0012; Tukey's *post hoc* analysis: CenA, PerA and PerF increased above baseline (p<0.002, p<0.001 and p<0.01 respectively).All behaviors were enhanced over those seen in the caffeine and the cocaine group.

NMI enables detection of neurochemicals and monitoring of behaviors *in vivo* before and after administration of cocaine in the same animal with no interruption in spatial or temporal resolution. This paradigm allows for “Same Animal Control” which provides more accurate results than was previously technically available. Figures 6, 7, 8, 9, 10, 11 and 12 are designed to show that neurochemical and behavioral changes after drug(s) administration are simultaneous. The format is as follows: **Neurochemical** effects of drug(s) on DA, 5-HT (A) and HVA and L-TP (B) in NAc of unrestained freely moving animals are shown by line graphs. Changes in current/concentration from baseline are reported on the y axes on the right (Baseline = 100%). **Behavioral** effects of drug(s) are shown in histogram form in (A) for comparison with DA and 5-HT and repeated in (B) for comparison with HVA and L-TP. Behavior is reported as frequency or number of times the animal crosses the infrared photobeams in log values on the y axes on the left (Baselines = Central Locomotion (Ambulation) *(CenA = 1.3)*; Peripheral Locomotion (Peripheral Ambulations) *(PerA=2.3)*; Stereotypy (Peripheral Fine Movements/Grooming) *(PerF = 2.0)*. Data are collected every five min and are presented in fifteen min intervals.

It is thought that acute cocaine and chronic administration produces its neurochemical and behavioral effects in brain reward centers by colocalized and interacting A2A/DA_2_ heteromeric GPRCs in NAc (6,48) and by mRNAs expression of main classes of G alpha proteins in NAc (49). Increased DA release derived from VTA may be a factor (6, 74). Chronic cocaine effects, on G protein signalling cascades, growth factors and other physiologic processes implicated in neuroplasticity in the DA reward mesolimbic pathway, are reviewed (50). Chronic cocaine responses to the extracellular signal-related kinase signalling pathway (ERK), brain derived neurotrophic factor (BDNF), glutamate transmission and synaptic plasticity are also discussed in the latter review.

### 3.2. Changes in neurochemicals and behavior after caffeine

Unlike cocaine, which exhibits a classical pharmacologic dose-response curve in which increasing doses of cocaine produce correlating increasing effects, caffeine exhibits an “Inverted U Shaped Function of Dose”. Due to this phenomenon, caffeine's effect on DA concentrations in NAc and DStr are reduced at high doses (50-100 mg/kg, ip), causing an aversive feeling, possibly by DA reduction and/or phosphodiesterase inhibition. The low dose of caffeine (about 3-15 mg/kg ip) and the moderate dose of caffeine (about 25-30 mg/kg ip) have a potent DA-ergic component and are reported to be euphoric and reinforcing in animals and humans (cf. 51 for review). In the present studies, we focus on the moderate dose of caffeine because our follow-up investigations after caffeine, consists of studies of caffeine and cocaine co-administered and at this moderate (middle) dose, caffeine potentiates cocaine-seeking behavior in animals (52).

Guanine-nucleotide-binding proteins (GP) are key components of cellular signalling. The first example of G Protein signalling, showing that a natural antagonist displays different affinities depending on which heteromer forms the targeted receptor, has been provided by caffeine. Adenosine A1 and A2A receptors form heteromers which alter the biochemical characteristics of the receptors, resulting, e.g., in a reduced affinity of the A1 receptor for agonists. These two receptors and their heteromers are expressed to a high degree in both pre-and post synaptic striatal neurons (cf 53 for review). Caffeine mechanisms have been shown to be mediated by a Gs protein/A2A antagonist receptor reaction(54). We selected the NAc-mesolimbic brain-reward pathway to study the psychostimulant properties of caffeine, wherein G Protein/D_1_-D_2_ dimers have been discovered (55).

NMI results, Figure 7A and B, show that caffeine, administered at a moderate dose, increases DA, 5-HT, HVA and L-TP and like cocaine, caffeine increases locomotion and stereotypy and reduces anxiety behavior. However, there are some interesting differences in the comparative results. Cocaine and caffeine effects on DA release in NAc parallel each other in the first hr; in the second hr, though, the DA response to caffeine is about 30% greater than cocaine. Serotonin release is greater in the caffeine group and L-TP release is dramatically greater in the caffeine group indicating that more L-TP is available for production of 5-HT in the caffeine group. Homovanillic acid responses to caffeine and cocaine are similar. Caffeine's effect on locomotion and anxiety reduction is greater than that of cocaine and caffeine's effect on stereotypy is greater than that of cocaine during the first hr.

Caffeine is a non-selective competitive blocker of A1 and A2A adenosine receptors (56,57). Caffeine is known to increase DA release in the mesolimbic and nigrostriatal pathways by A1 antagonism and caffeine is known to decrease movement behavior postsynaptically by A1 antagonism (8, 58). Since caffeine exerted enhanced DA release in mesolimbic NAc, we can assume that this occurred by adenosine A1 antagonist action at NAc nerve terminals. But, in these studies, movement behavior increased. Hence, we may further assume that the observed increased movement behavior at the post-synapse may occur by a different mechanism, possibly by a stimulatory action at A2A receptors in concert with D_2_ receptor heteromers, decreasing the affinity of the D_2_ receptor for DA and DA analogues (59). Moreover, the A1/A2A receptor heteromer is called a “sensor” for positive glutamate release, albeit in DStr, and high adenosine is correlated with prevailing A2A receptors which inhibit A1 receptor signalling *via* the heteromer (60).

### 3.3. Changes in neurochemicals and behavior after cocaine and caffeine in combination

We observed, after cocaine and caffeine were administered together, in tandem, to each animal, that all four neurochemicals and all three behaviors were not only dramatically increased above baseline but also significantly increased when comparisons were made between groups. The data are shown in Figure 8A and B.

The effects of caffeine/cocaine on DA release in NAc of the behaving animal supercedes those of cocaine alone by about 40% and 60% and those of caffeine alone by about 40% and 40% in the first and second hrs respectively. Serotonin and HVA signals increase about two-fold over caffeine and five fold over caffeine alone. L-Tryptophan responses in the combined caffeine/cocaine group were similar to those in the caffeine group but a four-fold increase occurred in this group compared with the cocaine group. Open-field behaviors also significantly increase over that produced by cocaine or caffeine alone. The cocaine/caffeine combination acts to a greater degree to reduce anxiety than does either cocaine or caffeine alone.

The observed dramatic enhancements of neurochemical and behavioral effects of co-administered cocaine and caffeine are likely due to the formation of heterotrimeric Gq/11 G proteins which derive from a different signalling mechanism mediated by the D_1_-D_2_ receptor heterodimer (dimer). In this dimer, Gq/11 protein forms in place of the Gi and the Gs coupled DA receptors, resulting in increased calcium mobilization, calmodulin kinase activation and hence, increased neurotransmitter-driven reward related behavior in NAc (53,55). The Gq/11 protein/DA mechanism has been reported to evoke neural and behavioral phenotypes with repeated cocaine administration (61). These dramatic enhancements of neurochemical and behavioral effects of co-administered cocaine and caffeine are probably also due to caffeine's ability to enhance cocaine-seeking at the moderate dose which, according to the “Inverted U-Shaped Function of Caffeine Dose”, exhibits the most potent DA response in NAc and DStr (51).

The present data are supported by previous data from this and other laboratories with a variety of different behavioral assays in which caffeine was studied at the moderate dose to enhance, increase or potentiate the effects of cocaine (62-65). Further evidence for attributing potentiated effects of caffeine on cocaine to caffeine's moderate dose, comes from data which show that low and high doses of caffeine are neuroprotective against cocaine-induced DA-ergic neurotoxicity (66,67).

A2A/D2 receptors form complex receptor heteromers that target G protein intracellular signalling cascades and play a role in control of movement, motor learning, motivation and brain reward mechanisms (68). We suggest that A2A/D2 heteromeric G-coupled complexes and/or colocalized and coexpressed D1/D2 Gq/11protein coupled dimers may mediate, at least in part, the potentiated neurochemical and behavioral responses observed when caffeine, at the moderate dose, is co-administered with cocaine.

### 3.4. Changes in neurochemicals and behavior after ketanserin

Our aim here is to use ketanserin to help delineate the mechanism of action of cocaine. The effects of ketanserin on neurochemicals and behavior are shown in Figure 9A and B.

As mentioned previously, there is a good rationale for the use of the antihypertensive, ketanserin, in this regard: (a) ketanserin is a direct receptor antagonist at 5-HT_2A/2C_ receptors, (b) based on the high presence of 5-HT_2A_ and 5-HT_2C_ receptors in NAc (69), ketanserin is most suitable for these studies because our biosensor is placed in NAc and (c) serotonin is an important neurotransmitter in brain reward (44,46) and in the antihypertensive action of ketanserin (70).

Results showed that DA, 5-HT, HVA and L-TP increase above baseline, but the frequency of behavioral events in the open-field paradigm are unremarkable after ketanserin injection. All four neurochemicals increase over cocaine except for DA release during the first hr. All four neurochemicals show lesser effects than caffeine except for the HVA response which shows an increase compared with caffeine during the second hr of study.

Direct 5-HT receptor antagonists are expected to increase 5-HT release due to an action on autoreceptors on presynaptic 5-HT cell bodies in dorsal raphe. Dopamine release in NAc is increased due to neuromodulation by 5-HT; the DA response to ketanserin is a secondary response. Is adenosine involved? This is unclear at this time. Nonetheless, ketanserin reverses catalepsy produced by DA_2_ antipsychotic agents with A2A agonist activity through 5-HT receptor activation (71). What is clear, though, is that the 5-HT_2A_ and 5-HT_2C_ receptors are Gq-coupled receptors. Indeed, treatment with the 5-HT agonist, (-)-1-(2,5-dimethoxy-4-iodophenyl)-2-aminopropane HCL (DOI), caused a down-regulation of 5-HT_2A_ receptors and a reduction in G-protein-coupled 5-HT_2A_ receptors. The authors suggest that DOI causes a phosphorylation of Galphaq/11protein and thereby contributes to the desensitization of 5-HT_2A_ receptors (72). Furthermore, ketanserin, itself, is reported to bind to G protein: guanosine-5′-(gamma-[(35)S]thio) triphosphate in several specific neuroanatomic substrates in brain and ketanserin was shown to bind to this G protein particularly in NAc (73).

### 3.5. Changes in neurochemicals and behavior after ketanserin and cocaine

All four neurochemicals and all three behaviors are attenuated by combining ketanserin with cocaine compared with results from cocaine alone. Dopamine, HVA and L-TP concentrations essentially show a return to baseline while 5-HT release is attenuated significantly below baseline. The data agree with previous studies wherein ketanserin antagonized locomotor behavior during cocaine challenge in a cocaine discrimination paradigm (74). The data also confirm previous studies from this laboratory wherein ketanserin attenuated the neurochemical and behavioral responses to cocaine (75). The data are shown in Figure 10A and B.

Cocaine increases blood pressure and heart rate primarily through an action on the sympathetic nervous system while suppressing baroreflexes and vagal tone; these actions further contribute to cocaine-induced tachycardia (76). Cocaine produces a pressor response associated with an initial hindquarters vasoconstriction followed by a prolonged vasodilation in conscious rats which has been reported to be antagonized by β adrenoreceptor antagonists like propranolol (77).

Ketanserin reduces blood pressure by 5-HT_2_ (70) and by 5-HT_2_ plus α_1_ adrenoreceptors (14). In sinoaortic denervated (SAD) rats and in spontaneously hypertensive rats (SHR), which have high blood pressure and high blood pressure variability, ketanserin lowered both cardiovascular parameters studied (78). Moreover, DA is related to the pathophysiology of hypertension. There are reports that DAD_1_ and DAD_2_ receptors are upregulated in DStr of young, prehypertensive rats and cholecystokinin 8S-induced release of DA in NAc is greater in SHR rats than in normals (79,80).

Therefore, ketanserin may be attenuating cocaine effects through its 5-HT-ergic and DA-ergic antihypertensive effects as well as through its GPRC-ligand related functional selectivity. Indeed, recent studies in humans, using Positron Emission Tomography, have shown a high correlation between increased DA in DStr and increased blood pressure after administration of the cocaine-like psychostimulant, methylphenidate (81). We suggest that the antihypertensive properties of ketanserin provide a reasonable mechanism for attenuating, at least in part, psychostimulant monoamine release and behavior produced by cocaine; the mechanism may occur through a 5-HT_2A/2C_ modulated DA_2_ response to cocaine with a significant role played by the family of the GPs and their heteromers.

### 3.6. Changes in neurochemicals and behavior after ketanserin and caffeine

Only three of four neurochemicals and all three open-field behaviors produced by caffeine are attenuated by ketanserin. The data are shown in Figure 11A and B. Ketanserin did not inhibit DA release in NAc. On the contrary, DA release is enhanced in NAc when ketanserin and caffeine are administered together. This enhancement of DA release in NAc is surprising as unlike ketanserin/cocaine results, brain reward mechanisms and hypertension appear to be only partially inhibited. Also, it is noteworthy that enhanced DA release in NAc after ketanserin and caffeine administration could have adverse medical implications, since it is known that the pathophysiology of hypertension in SHR animals involves an increase in DA in NAc and DStr.

Enhancement in the DA response in NAc in this group is seen in the second hr; the DA response to ketanserin/caffeine is enhanced over caffeine alone by more than 30% and actually matches the cocaine/caffeine effect on DA release in NAc. The HVA response to ketanserin/caffeine is similar to that of caffeine and the L-TP response is blocked as compared to caffeine effects when caffeine is administered alone. The 5-HT response produced by caffeine is dramatically inhibited by ketanserin by about 70%. All open-field behaviors, which increase after caffeine, are attenuated by ketanserin.

Ketanserin may be affecting brain reward and hypertensive mechanisms through its 5-HT-ergic and DA-ergic effects and downstream through GPRCs, cyclases, and kinases. Given the caveat that these studies are acute experiments which involve a single injection of ketanserin and caffeine, the data may indicate adverse reactions only to **acute** ketanserin and caffeine. Therefore, the data may not predict that adverse reactions to ketanserin and caffeine will occur during chronic administration of these compounds.

### 3.7. Changes in neurochemicals and behavior after ketanserin, cocaine and caffeine

These data are shown in Figure 12 A and B. When caffeine is co-administered with ketanserin and cocaine, ketanserin continues to inhibit 5-HT release and production of 5-HT through L-TP synthesis. The HVA response is inhibited to below baseline. But, DA release is enhanced as in the ketanserin/caffeine group. Like the ketanserin/caffeine effect, DA release reached that observed in the cocaine/caffeine group. As mentioned previously, enhancement of DA release in NAc could be worrisome as antihypertensive action has been correlated with decreased and not increased DA concentrations in NAc and DStr (79,80). The main difference between this group and the ketanserin/caffeine group lies in the open-field behavioral responses. Open-field behaviors are not blocked by ketanserin; instead, open-field movement behaviors are actually increased.

The data show that ketanserin is unable to block the DA neurochemical effects of cocaine when caffeine is present and ketanserin is unable to block DA effects of caffeine whether or not cocaine is present. The mechanism for these findings is not yet known. However, it is likely that blockade of G-coupled 5-HT_2A2C_ receptors by ketanserin neuromodulates enhanced DA release in NAc. The increase in open-field behaviors, observed in this group insofar as at least increased locomotion is concerned, may derive from postsynaptic A2A/DA_2_ heteromers and/or from DA-ergic release presynaptically in VTA, similarly to what happens when cocaine affects locomotor behavior.

**Figure 13.**
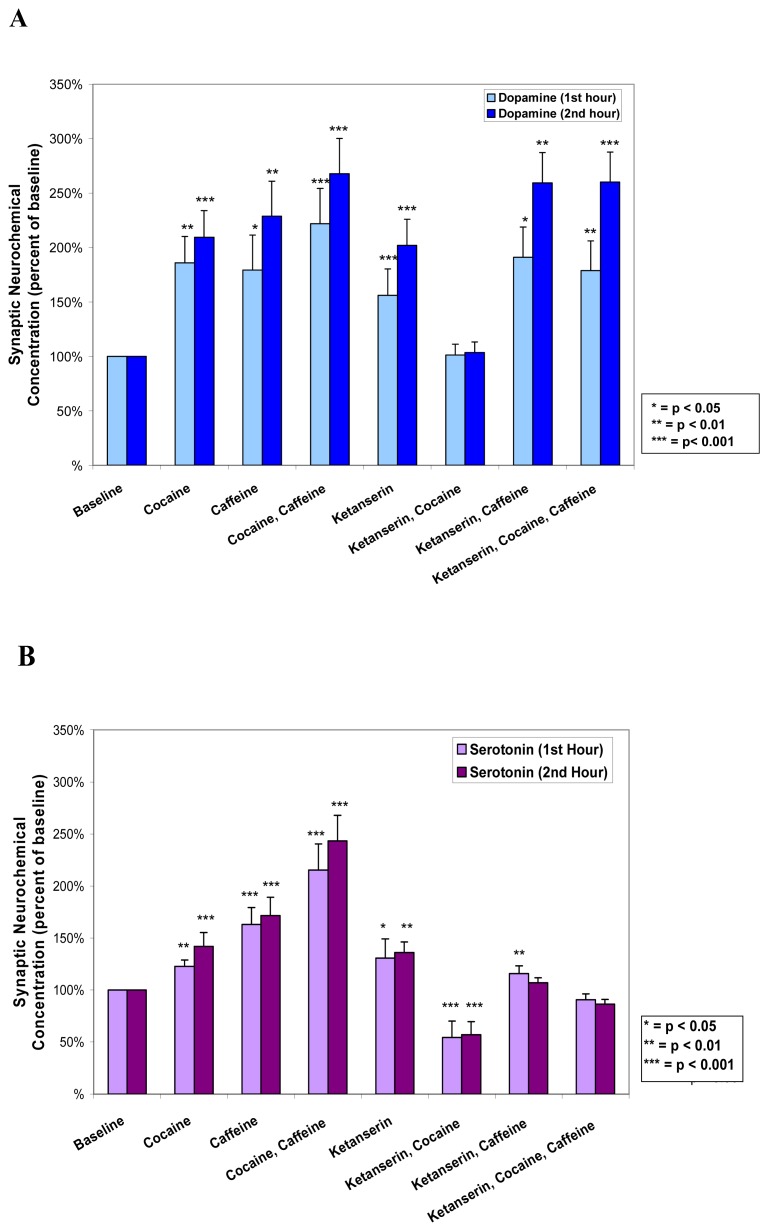
**Figure 13A and B** shows histograms derived from hourly effects of drug(s) on DA and 5-HT release in NAc in unrestrained animals.

The composite histograms, (Figure 13A and B), depicting the neurochemical effects of each set of the drug(s) studied herein, further clarify the dynamic relationship between DA and 5-HT release within NAc in unrestrained animals. The potentiation of the moderate dose of caffeine on cocaine monoamine responses is clear. The direct receptor 5-HT-ergic antagonist effect of ketanserin, *per se*, on the monoamines is also clear. That ketanserin inhibits cocaine-induced monoamines and inhibits only the caffeine-induced 5-HT-ergic amine aspect, is clear. Thus, ketanserin's effects on cocaine and caffeine, although both psychostimulants, are different. All NMI studies are performed within ventrolateral NAc. Since NMI biosensors are known for precise spatial resolution, we can assume that the same neuronal tissue is imaged. We can reasonably assume, then, that 5-HT-G-coupled proteins are acting in concert with DA and Adenosine G-coupled receptors to differentiate the neuronal mechanisms of cocaine, caffeine and ketanserin in NAc.

Binding to the intracellular loops of receptors, these GPRCs and their effects on intracellular and extracellular signalling are providing new targets for psychostimulant therapeutics. Cocaine has been shown to act on G proteins and on signalling cascades in NAc, PFC and DStr (82-84) and G proteins have been implicated in the effects of prenatal exposure to cocaine (85). Downstream in the cascade, DA and cAMP-regulated phosphoprotein, Mr 32 kDa (DARPP-32) has been identified as a major target for DA and protein kinase A (PKA) and recently, the regulation of the specific state of DARPP-32 phosphorylation has provided an integrated mechanism for the study of dopaminoceptive neurons; both cocaine and caffeine act via DARPP-32 (cf 86 for review).

Particularly for ketanserin, ligand-directed functional selectivity as well as GPRCs, may have significant input. Thus, “cross-talk” between and among these cellular pathways may well underlie differential, unexpected results during co-administration of these psychostimulants with ketanserin. We plan to add molecular studies of the G-protein family and signalling cascades to our studies of ketanserin and psychostimulant interactions.

## 4. Conclusions

With NMI and BRODERICK PROBE^®^ biosensors, we analyzed (a) brain mechanisms of psychostimulants *in vivo*, (b) pharmacologic treatments to attenuate the symptoms of substance abuse, (c) critical concentration effects of caffeine and (d) possible untoward side effects of medicinal compounds used with caffeine. The performance of these studies has not been possible with *previous* technologies. The animal studies, presented here, are highly contributive to science and medicine. Furthermore, we are in the midst of applying our biosensors in animal studies to decipher neurochemical mechanisms which may cause Parkinson's disease (87), cognitive dysfunction post-operatively (88), hypotension and stroke.

Yet, all of the aforementioned studies represent only a fascinating prelude to the promise of clinical trials using the BRODERICK PROBE^®^ biosensors to diagnose epilepsy and tumor disorders in patients. In fact, Drs. Kuzniecky, Doyle, Pacia, Li and Broderick will use these inventive biosensors to image neurotransmitters within the neocortex of patients intraoperatively with approval from the Internal Review Board of the New York University (NYU) School of Medicine.
